# The quest to identify the mechanism underlying adrenergic regulation of cardiac Ca^2+^ channels

**DOI:** 10.1080/19336950.2020.1740502

**Published:** 2020-03-20

**Authors:** Daniel Roybal, Jessica A. Hennessey, Steven O. Marx

**Affiliations:** a Division of Cardiology, Department of Medicine, Columbia University, Vagelos College of Physicians and Surgeons, New York, USA; b Department of Pharmacology, Columbia University, Vagelos College of Physicians and Surgeons

**Keywords:** Calcium channel, adrenergic regulation, phosphorylation, protein kinase A, heart

## Abstract

Activation of protein kinase A by cyclic AMP results in a multi-fold upregulation of Ca_V_1.2 currents in the heart, as originally reported in the 1970's and 1980's. Despite considerable interest and much investment, the molecular mechanisms responsible for this signature modulation remained stubbornly elusive for over 40 years. A key manifestation of this lack of understanding is that while this regulation is readily apparent in heart cells, it has not been possible to reconstitute it in heterologous expression systems. In this review, we describe the efforts of many investigators over the past decades to identify the mechanisms responsible for the β-adrenergic mediated activation of voltage-gated Ca^2+^ channels in the heart and other tissues.

The phrase “fight or flight” was coined by Walter Bradford Cannon at Harvard Medical School in 1915 in his book *Bodily Changes in Pain, Hunger, Fear and Rage* [1]. He described how both physical trauma and psychological emergencies have the same effects on the body through the release of catecholamines (e.g. epinephrine and norepinephrine) into the bloodstream. He further described some of those effects including dilation of skeletal muscle blood vessels, constriction of skin blood vessels, and release of glucose into the bloodstream. Additionally, he and others described the physiologic effects on the heart, including increased heart rate, contractility and relaxation (chronotropy, inotropy and lusitropy, respectively). It was not until the discovery of the β-adrenergic receptor in the mid 1980's by Robert Lefkowitz and his colleagues [2] that we began to understand how epinephrine and norepinephrine are able to exert those effects on the heart. The heart contains β_1_ and β_2_ adrenergic receptors. These G-proteincoupled receptors, once bound by epinephrine or norepinephrine, activate a G-protein signaling cascade that ends in activation of protein kinase A (PKA), which phosphorylates a number of proteins involved in excitation-contraction coupling to increase chronotropy, inotropy and lusitropy.

The cardiac L-type Ca^2+^ channel, also known as Ca_V_1.2, plays a critical role in excitation-contraction coupling. The Ca_V_1.2 channels are characterized by their high voltage-activating and slow inactivating properties. They contain at minimum a pore-forming subunit (α_1__C_) with 4 homologous transmembrane domains consisting of 6 transmembrane segments with a membrane-associated pore loop between transmembrane segments 5 and 6, and cytoplasmic N- and C-termini (Figure 1(a)). An auxiliary β subunit (β_2B_ in heart) binds to the α-interacting domain in the I–II loop with high affinity (Figure 1(a)) and plays roles in surface expression of α_1__C_ and channel availability [3]. Normal cardiac excitation-contraction coupling occurs when initiation of the cardiac action potential activates Ca_V_1.2 channels, which reside in the transverse tubules (T-tubules) of the cardiomyocyte [4–6]. This leads to Ca^2+^-induced Ca^2+^ release whereby the ryanodine receptor releases Ca^2+^ from the sarcoplasmic reticulum (SR). This Ca^2+^ then binds troponin C, permitting the crosslinking of myofilaments. The majority of the Ca^2+^ is then removed from the cytosol via the sarcoplasmic-endoplasmic reticulum Ca^2+^-ATPase (SERCA), modulated by phospholamban (PLB), and the Na^+^-Ca^2+^ exchanger.

**Figure 1.**
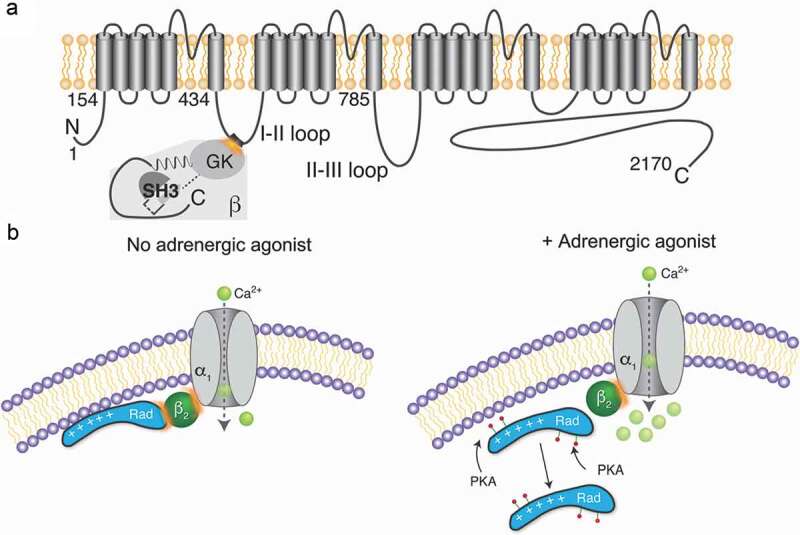
Schematic of Ca_V_1.2 and proposed model of β-adrenergic regulation of Ca_V_1.2. (a) Diagram showing rabbit cardiac α_1__C_ and β_2B_ subunits. GK, guanylate kinase domain; SH3, Src homology 3 domain. (b) No adrenergic agonist – basal state model (left): Rad associates with Ca_V_1.2 β subunit and the plasma membrane, thereby inhibiting channel activity. Adrenergic agonist – stimulated state model (right): PKA phosphorylation of Rad reduces the affinity of Rad with the membrane and with the Ca_V_β subunit. Phosphorylated Rad leaves the “neighborhood” of the Ca^2+^ channel resulting in increased Ca^2+^ influx (green circles). Adapted from [59].

Upon activation of the β-adrenergic signaling cascade, PKA is activated and directly phosphorylates several targets in the heart including the ryanodine receptor [7], troponin-I [8] and phospholamban [9], leading to increased Ca^2+^ release from the SR and thereby increased myofilament cross-linking, increased Ca^2+^ re-uptake into the SR, and increased myofilament relaxation. Importantly, activation of β-adrenergic receptors also leads to an increase in Ca_V_1.2 current and availability [10,11]. The mechanism through which β-adrenergic signaling exerts this effect has been the subject of debate. Activation of PKA is a universally accepted requirement for regulation of the channel [12–14], but relevant functional target sites have continued to been elusive for more than 40 years [15]. Although the obvious targets of phosphorylation are Ca_V_1.2 α or β subunits (Figure 1(a)) which have been the focus of most major studies to date, the data have been contradictory and controversial. Full reconstitution of the β-adrenergic receptor signaling pathway in heterologous expression systems remained an unmet challenge [16] implying critical gaps in our understanding of the process. Here, we provide a historical perspective on the quest to discover the mechanisms of adrenergic regulation of cardiac Ca_V_1.2, and describe how new technology led to a shift in that paradigm.

## Heterologous reconstitution of adrenergic regulation

Numerous attempts have been made to reconstitute adrenergic regulation of Ca_V_1.2 by PKA activation in heterologous expression systems, done in a variety of models including *Xenopus* oocytes and various cell lines. Early attempts at reconstitution included work by Kameyama and Nakayama who solubilized cardiac Ca^2+^ channels into artificial lipid bilayers after mixing purified sarcolemma and membrane proteins with soybean phospholipid to form proteoliposomes [17]. They demonstrated that Ca^2+^ current from proteoliposomes incubated with activated PKA and Mg^2+^-ATP was 5 times larger than the controls, implying that activation of Ca_V_1.2 occurs via direct phosphorylation of a channel subunit or a protein that remains closely associated with the channel. In oocytes, reconstitution of adrenergic regulation of Ca_V_1.2 was accomplished by injection of total cardiac mRNA [18]. The injected mRNA encoded not only the α_1__C_ subunit, but also other protein(s) that could enable PKA regulation of Ca_V_1.2 to occur. In retrospect, these studies may have revealed the importance of an as of yet unidentified protein in the regulation of Ca_V_1.2 current. Other studies reported successful reconstitution simply using α_1__C_ and/or β subunits [19–22]. The concept that AKAPs, which bind and sequester PKA to specific subcellular locations, was also considered in these studies [23–26].

The above results were not without some controversy as researchers had difficulty reproducing reconstitution results. In an attempt to categorically identify essential protein components, *Xenopus* oocytes expressing cardiac Ca_V_1.2 channels with various combinations of auxiliary subunits were studied [27]. No increase in current through α_1__C_ was shown after addition of cAMP or the catalytic subunit of PKA. Perez-Reyes et al. showed that only after PKA inhibition by H-89, a forskolin-mediated increase in current was observed in HEK cells co-transfected α_1_ and β_2A_ subunits. This suggested a basal-state phosphorylation of the expressed channel or an associated protein in these cells [28]. In a comprehensive study of both CHO cells stably expressing α_1__C_ and HEK cells transiently expressing α_1__C_ and β_2_ subunits, neither inhibition of endogenous PKA, inhibition of protein phosphatases, or internal dialysis of PKA significantly affected peak barium current through α_1__C_ [29]. Mikala et al. suggested that α_1__C_ is unable to be regulated by phosphorylation in HEK cells after showing that a high concentration of H-89, a high concentration of the catalytic subunit of PKA, or 8-Br-cAMP had no effect on the activity of the wild-type channel [30].

A role for proteolytic cleavage of the C-terminus of α_1__C_ was also proposed as an essential process for adrenergic signaling. Different sizes of α_1__C_ have been detected in skeletal muscle [31,32], brain [33,34], and cardiac sarcolemmal membranes [35]. Truncation of 46% to 70% of the C-terminus of α_1__C_ results in increased activity of the channel [36], suggesting an inhibitory activity of this region. Cleavage of the C-terminus of α_1__C_ was proposed to be essential for PKA regulation of the channel because the release of the autoinhibitory action of the C-terminus by phosphorylation of Ser1700 was required [37]. Without cleavage, release of the autoinhibition could not occur. In the heart, however, C-terminal cleavage is absolutely not required for β-adrenergic stimulation of Ca_V_1.2. We showed this by generating transgenic mice with inducible expression of proteolytic-resistant α_1__C_ [38].

## Identifying and testing PKA phosphorylation sites

After the cloning of α_1__C_ [39], the sequence was subsequently analyzed for putative phosphorylation sites. Early site-specific identification efforts showed potential PKA phosphorylation of Ser1627 and Ser1700 [40]. Other suggested sites of regulation included Ser1829 [41] and Ser1142 [42] and Ser1928 [35,43]. The concept that Ser1928 mediates activation of Ca_V_1.2 was further supported through heterologous expression of a Ser1928 mutant that prevented upregulation of Ca_V_1.2 [23]. Other investigators failed to reproduce PKA modulation of Ca^2+^ currents in heterologous expression systems [29,30]. Phosphorylation sites have also been mapped to the Ca^2+^ channel β subunit at Ser459, Ser478 and Ser479 [44–46].

Given the challenges associated with heterologous expression systems and improvements in the development of genetically-altered mice, many investigators turned to ventricular myocytes to study mechanisms responsible for adrenergic regulation of Ca_V_1.2. The dogma that phosphorylation of Ser1928 by PKA is required for β-adrenergic stimulation of Ca_V_1.2 was tested by the O’Rourke group in 2006 through adenoviral gene transfer to express mutant α_1__C_ subunits in guinea pig cardiomyocytes [47]. Interestingly, an alanine point mutation at Ser1928 did not significantly reduce the response of α_1__C_ to isoproterenol. Two years later, Lemke et al. confirmed that phosphorylation of Ser1928 is not required for adrenergic regulation of Ca_V_1.2 via generating a knock-in mouse with an alanine substitution at Ser1928 [48].

To test whether PKA phosphorylation of the β subunit was relevant in cardiomyocytes, β subunit with alanine substitutions at the three putative phosphorylation sites was expressed in cardiomyocytes. These sites were shown to be unessential for adrenergic stimulation of Ca_V_1.2 [47,49]. As definitive proof that these sites were not required, a knock-in mouse expressing a β_2_ subunit truncated prior to these phosphorylation sites displayed normal PKA modulation of Ca_V_1.2, thus ruling out involvement of any putative C-terminal phosphorylation sites in mediating β-adrenergic regulation of Ca_V_1.2 [50].

As a heteromultimeric complex, Ca_V_1.2 natively associates with a variety of auxiliary proteins, and several of these proteins have the ability of modulating channel activity. Neuroblast differentiation-associated protein ahnak has been shown to associate with Ca_V_β_2_ and this association decreases after PKA phosphorylation of both ahnak and the β subunit [51]. While it was initially proposed that phosphorylation and subsequent release of ahnak from β_2_ allowed for greater binding of β_2_ to α_1__C_ leading to enhanced Ca^2+^ current during adrenergic activation, later studies showed that ahnak-deficient cardiomyocytes displayed preserved adrenergic modulation of Ca_V_1.2 [52].

## Additional phosphorylation sites

After phosphorylation of Ser1928 was shown to be non-essential for β-adrenergic regulation of Ca_V_1.2 in the heart, phosphorylation of Ser1700 and Thr1704 was proposed [37,53]. To expedite the process of studying multiple phosphorylation sites in mice, we developed a transgenic approach that enabled doxycycline-inducible expression of FLAG-tagged, dihydropyridine (DHP)-resistant Ca_V_1.2 channels in mice [54]. The transgenic and endogenous Ca_V_1.2 currents are distinguishable by application of nisoldipine, a Ca^2+^ channel dihydropyridine-antagonist [54]. This approach not only circumvented any potential developmental abnormalities caused by constitutive expression of the transgene, but also prevented high levels of basal transgenic current which might mask any observed adrenergic regulation of the channel [49,55]. We first generated transgenic mice with inducible expression of a DHP-resistant (T1066Y/Q1070M) N-terminal 3X FLAG-epitope-tagged α_1__C_, designated pseudo-WT (pWT α_1__C_). The resultant DHP-resistant Ca^2+^ current responded normally to adrenergic stimulation, nearly doubling after superfusion of isoproterenol in cardiomyocytes. By creating additional transgenic mice with alanine-substitutions of Ser1700 and Thr1704, residues proposed as essential phosphorylation sites based on experiments in tsA-201 cells [37], we found that phosphorylation of Ser1700 and Thr1704 is not required [54].

Independently, the Catterall group generated a mutant knock-in mouse line with alanine substitutions at Ser1700 and Thr1704 [56]. They reported that phosphorylation of Ser1700 alone or in combination with Thr1704 is required, basing their conclusion upon an unconventional metric: the difference in absolute current amplitude rather than the fold-increase after isoproterenol, which is the standard analysis. Their metric is valid only if the density of Ca^2+^ channels at the surface was unchanged, yet basal Ca^2+^ currents were substantially reduced [56,57]. Hofmann and colleagues subsequently created S1700A/T1704A knock-in mice and concluded that isoproterenol stimulated Ca^2+^ current in the control and mutant S1700A/T1704A cardiomyocytes to the same extent [58]. Furthermore, Hofmann’s group recalculated Catterall’s data and showed that in both groups’ knock-in mice, the β-adrenergic stimulation for wild-type and mutant channels were equivalent [58]. This confirmed our initial findings [54], which we have further substantiated with additional transgenic mice [59,60].

The failure to find any single α_1__C_ site as essential for β-adrenergic modulation of Ca_V_1.2 led us to hypothesize that a combination of sites in α_1__C_ is required. After identifying conserved PKA consensus sequences in α_1__C_ of five species (mouse, rat, rabbit, guinea pig and human), we generated α_1__C_ transgenic mice (“22-mutant α_1__C_”) in which we replaced 17 conserved consensus PKA phosphorylation sites that were not previously studied, and 5 conserved PKA/CaMKII phosphorylation sites known to be non-essential including Ser1700 and Thr1704 [35,47,50,54]. Surprisingly, we found that none were necessary [38]. This led us to then hypothesize that the functionally-relevant PKA targets in α_1__C_ might be different amongst these five species. To test this, we mutated all 51 conserved and non-conserved serine and threonine residues within the 35 intracellular PKA consensus phosphorylation sites of rabbit α_1__C_ to alanine (“35-mutant α_1__C_”). In cardiomyocytes, the nisoldipine-insensitive 35-mutant Ca^2+^ currents were both up-regulated and activated at more negative potentials in response to isoproterenol or forskolin [59].

Similarly, we mutated to alanine all 37 conserved and non-conserved serine and threonine residues within 28 PKA-consensus phosphorylation sites of human β_2B_ (“28-mutant β_2B_”). Cardiomyocytes expressing GFP-tagged 28-mutant β_2B_ displayed isoproterenol- or forskolin-induced stimulation of Ca_V_1.2 current amplitude. Finally, we crossed 35-mutant α_1__C_ with 28-mutant β_2B_ transgenic mice. These mutant channels also displayed a normal isoproterenol- or forskolin-induced increase in peak Ca^2+^ current [59]. These results indicate that β-adrenergic stimulation of Ca_V_1.2 does not involve direct phosphorylation of α_1__C_ or β_2_ subunits.

## Rad is the PKA target

Cognizant of the possibility that an unknown protein may mediate adrenergic regulation, we took an unbiased approach and sought to determine the comprehensive interactome of Ca_V_1.2 in the heart. Proteomic analysis of membrane-signaling complexes from native tissue by traditional means alone is challenging, however, as many interactions are labile under harsh purification conditions. We turned to proximity labeling and quantitative proteomics [61–63]. As channel regulation only reliably manifests in native tissues, we extended this method to native tissues and whole organs of transgenic mice. We generated transgenic mice with doxycycline-inducible, cardiomyocyte-specific expression of DHP-resistant-α_1__C_ or β_2B_ proteins with ascorbate-peroxidase (APEX2) and a V5 epitope conjugated to the N-termini, enabling biotin-labeling of proteins within ~20 nm of the Ca^2+^ channels [59]. After exposure to isoproterenol, the enrichment of Rad, a member of the Rad and Gem/Kir Ras-related GTP-binding protein (RGK) family of GTP-binding proteins known for their capacity to inhibit all high-voltage-activated Ca^2+^ channels [64,65], was decreased from the neighborhood of Ca_V_1.2.

Rad-knockout mice display an increased maximum Ca^2+^ current, and the Ca^2+^ channels activate at lower voltages, mimicking the effects of β-adrenergic receptor stimulation [66,67]. Other studies, however, led to expectations that Rad is not directly involved in adrenergic regulation of Ca_V_1.2 since adenoviral-induced overexpression of Rad [68] and Rem [69] in cultured cardiomyocytes ablated Ca_V_1.2 and attenuated adrenergic regulation. The proximity labeling experiments suggested, however, that Rad was the missing link.

The robust heterologous reconstitution of PKA regulation of Ca_V_1.2 currents has been long pursued as a crucial step in identifying and validating the mechanism. To reconstitute PKA regulation in a heterologous expression system, we carefully considered experimental conditions. We used perforated patch clamp techniques, to preserve the intracellular milieu, and a voltage ramp, rather than step protocols, which required the use of Ba^2+^ rather than Ca^2+^ to minimize inactivation [59]. A step protocol, however, was also effective. Perhaps most importantly, we co-expressed a limited amount of Rad with α_1__C_ and β_2b_ subunits in HEK293 T cells, adding 3- to 6-fold less Rad than Ca_V_1.2 subunits. We reasoned that excess Rad could eliminate Ca^2+^ current.

In HEK293T cells transfected with only α_1__C_ plus β_2B_, superfusion of forskolin over 1–3 minutes had no impact on Ba^2+^ current. Applying forskolin to cells expressing α_1__C_ with β_2B_ and Rad, in contrast, increased the maximal conductance and shifted the *V*_50_ for activation. Forskolin also increased the maximal conductance of Ca_V_1.2 channels comprised of 35-mutant α_1__C_ with 28-mutant β_2B_ and Rad, implying that similar to cardiomyocytes, phosphorylation of α_1__C_ and β_2B_ subunits is not required [59]. Rad is also a potential PKA target [70]. Using mass spectrometry, we identified three phosphorylation sites on Rad: Ser25, Ser38 and Ser300. We were unable to detect non-phosphorylated or phosphorylated peptides containing Ser272, but we presumed that it was also a PKA phosphorylation site based upon prior biochemical studies [70]. Alanine-substitutions of these four serine residues in Rad prevented the forskolin-induced increase in maximal conductance. We found that alanine substitutions at Ser272 and Ser300 within the C-terminal polybasic membrane region of Rad prevented both the forskolin-induced increase in current amplitude and the hyperpolarizing shift in *V*_50_ [59].

Rad can inhibit Ca_V_1.2 via Ca_V_β-binding-dependent and independent (α_1__C_-dependent) mechanisms [71,72]. Mutating residues on Rad or on β_2B_ that prevented interaction between Rad and β_2B_ [71,73] prevented the forskolin-induced increase in the maximal conductance and the hyperpolarizing shift in the I–V curve. Thus, the interaction between Rad and β subunits is essential for cAMP–PKA regulation of Ca_V_1.2 in HEK cells [59]. These results are consistent with our findings that the β subunit association with α_1__C_ is absolutely required for β-adrenergic stimulation of Ca_V_1.2 currents in cardiomyocytes [60].

## Conclusions

In cardiomyocytes, β-adrenergic signaling stimulates cAMP production and subsequently activates PKA. PKA phosphorylation of Rad leads to its displacement from Ca_V_β, thereby releasing the channel from an inhibited state (Figure 1(b)). The PKA-mediated phosphorylation of an inhibitor and subsequent release of the inhibitor from the complex is reminiscent of the effect of PKA phosphorylation of phospholamban on SERCA [74]. The mechanism appears to be transferable to other voltage-gated Ca^2+^ channels, such as Ca_V_2.2 and Ca_V_1.3, which also bind β subunits, and are regulated by forskolin when Rad or Rem are co-expressed [59]. We hypothesize that phosphorylation of the C-terminus of Rad (or Rem) alters the electrostatic interactions with the plasma membrane, as well as the interactions with β subunits, thereby releasing the channel from an inhibited state. The identification of this mechanism could offer opportunities to develop specific modulators of the sympathetic nervous system modulation of Ca^2+^ currents for patients with heart failure for instance. Future projects will also explore the role of Rad phosphorylation using phospho-mutant knock-in mice, and how the neighborhood of the Ca^2+^ channels change in disease states.

## References

[1] Cannon WB. Bodily changes in pain, hunger, fear, and rage: an account of recent researches into the function of emotional excitement. New York, NY: D Appleton & Company; 1915.

[2] Dixon RA, Kobilka BK, Strader DJ, et al. Cloning of the gene and cDNA for mammalian beta-adrenergic receptor and homology with rhodopsin. Nature. 1986;321:75–79. 3010132 10.1038/321075a0

[3] Lacerda AE, Kim HS, Ruth P, et al. Normalization of current kinetics by interaction between the alpha 1 and beta subunits of the skeletal muscle dihydropyridine-sensitive Ca2+ channel. Nature. 1991;352(6335):527–530. 1650913 10.1038/352527a0

[4] Fabiato A, Fabiato F. Excitation-contraction coupling of isolated cardiac fibers with disrupted or closed sarcolemmas: calcium-dependent cyclic and tonic contractions. Circ Res. 1972;31(3):293–307. 4341466 10.1161/01.res.31.3.293

[5] Fabiato F, Fabiato A. Excitation-contraction coupling and regulation of contractility studied on isolated adult myocardial cells. Acta Cardiol. 1972;27(2):243–248. 4557640

[6] Bers DM. Cardiac excitation-contraction coupling. Nature. 2002;415(6868):198–205. 11805843 10.1038/415198a

[7] Marx SO, Reiken S, Hisamatsu Y, et al. PKA phosphorylation dissociates FKBP12.6 from the calcium release channel (ryanodine receptor): defective regulation in failing hearts. Cell. 2000;101(4):365–376. 10830164 10.1016/s0092-8674(00)80847-8

[8] Rao V, Cheng Y, Lindert S, et al. PKA phosphorylation of cardiac troponin i modulates activation and relaxation kinetics of ventricular myofibrils. Biophys J. 2014;107(5):1196–1204. 25185555 10.1016/j.bpj.2014.07.027 PMC4156663

[9] Kirchberber MA, Tada M, Katz AM. Phospholamban: a regulatory protein of the cardiac sarcoplasmic reticulum. Recent Adv Stud Cardiac Struct Metab. 1975;5:103–115. 127351

[10] Reuter H. The dependence of slow inward current in Purkinje fibres on the extracellular calcium-concentration. J Physiol. 1967;192:479–492. 6050160 10.1113/jphysiol.1967.sp008310 PMC1365567

[11] Vassort G, Rougier O, Garnier D, et al. Effects of adrenaline on membrane inward currents during the cardiac action potential. Pflugers Arch. 1969;309:70–81. 5815320 10.1007/BF00592283

[12] Kamp TJ, Hell JW. Regulation of cardiac L-type calcium channels by protein kinase A and protein kinase C. Circ Res. 2000;87:1095–1102. 11110765 10.1161/01.res.87.12.1095

[13] Tsien RW. Calcium channels in excitable cell membranes. Annu Rev Physiol. 1983;45(1):341–358. 6303205 10.1146/annurev.ph.45.030183.002013

[14] Trautwein W, Hescheler J. Regulation of cardiac L-type calcium current by phosphorylation and G proteins. Annu Rev Physiol. 1990;52(1):257–274. 2158764 10.1146/annurev.ph.52.030190.001353

[15] Wang X, Tsien RW. Suspect that modulates the heartbeat is ensnared. Nature. 2020;577(7792):624–626. 31988403 10.1038/d41586-020-00096-3

[16] Weiss S, Oz S, Benmocha A, et al. Regulation of cardiac L-type Ca(2)(+) channel CaV1.2 via the beta-adrenergic-cAMP-protein kinase A pathway: old dogmas, advances, and new uncertainties. Circ Res. 2013;113:617–631. 23948586 10.1161/CIRCRESAHA.113.301781

[17] Kameyama A, Nakayama T. Calcium efflux through cardiac calcium channels reconstituted into liposomes–flux measurement with fura-2. Biochem Biophys Res Commun. 1988;154(3):1067–1074. 2457364 10.1016/0006-291x(88)90249-5

[18] Lory P, Nargeot J. Cyclic AMP-dependent modulation of cardiac Ca channels expressed in Xenopus laevis oocytes. Biochem Biophys Res Commun. 1992;182(3):1059–1065. 1371671 10.1016/0006-291x(92)91839-i

[19] Sculptoreanu A, Rotman E, Takahashi M, et al. Voltage-dependent potentiation of the activity of cardiac L-type calcium channel alpha 1 subunits due to phosphorylation by cAMP-dependent protein kinase. Proc Natl Acad Sci U S A. 1993;90(21):10135–10139. 7694283 10.1073/pnas.90.21.10135 PMC47728

[20] Nunoki K, Florio V, Catterall WA. Activation of purified calcium channels by stoichiometric protein phosphorylation. Proc Natl Acad Sci U S A. 1989;86(17):6816–6820. 2549550 10.1073/pnas.86.17.6816 PMC297937

[21] Chang CF, Gutierrez LM, Mundina-Weilenmann C, et al. Dihydropyridine-sensitive calcium channels from skeletal muscle. II. Functional effects of differential phosphorylation of channel subunits. J Biol Chem. 1991;266(25):16395–16400. 1653234

[22] Yoshida A, Takahashi M, Nishimura S, et al. Cyclic AMP-dependent phosphorylation and regulation of the cardiac dihydropyridine-sensitive Ca channel. FEBS Lett. 1992;309(3):343–349. 1325377 10.1016/0014-5793(92)80804-p

[23] Gao T, Yatani A, Dell’Acqua ML, et al. cAMP-dependent regulation of cardiac L-type Ca2+ channels requires membrane targeting of PKA and phosphorylation of channel subunits. Neuron. 1997;19(1):185–196. 9247274 10.1016/s0896-6273(00)80358-x

[24] Gray PC, Johnson BD, Westenbroek RE, et al. Primary structure and function of an A kinase anchoring protein associated with calcium channels. Neuron. 1998;20(5):1017–1026. 9620705 10.1016/s0896-6273(00)80482-1

[25] Gray PC, Tibbs VC, Catterall WA, et al. Identification of a 15-kDa cAMP-dependent protein kinase-anchoring protein associated with skeletal muscle L-type calcium channels. J Biol Chem. 1997;272(10):6297–6302. 9045648 10.1074/jbc.272.10.6297

[26] Fraser ID, Tavalin SJ, Lester LB, et al. A novel lipid-anchored A-kinase Anchoring Protein facilitates cAMP-responsive membrane events. Embo J. 1998;17:2261–2272. 9545239 10.1093/emboj/17.8.2261 PMC1170570

[27] Singer-Lahat D, Lotan I, Biel M, et al. Cardiac calcium channels expressed in Xenopus oocytes are modulated by dephosphorylation but not by cAMP-dependent phosphorylation. Recept Channels. 1994;2(3):215–226. 7874448

[28] Perez-Reyes E, Yuan W, Wei X, et al. Regulation of the cloned L-type cardiac calcium channel by cyclic-AMP-dependent protein kinase. FEBS Lett. 1994;342(2):119–123. 8143862 10.1016/0014-5793(94)80484-2

[29] Zong X, Schreieck J, Mehrke G, et al. On the regulation of the expressed L-type calcium channel by cAMP-dependent phosphorylation. Pflugers Arch. 1995;430(3):340–347. 7491257 10.1007/BF00373908

[30] Mikala G, Klockner U, Varadi M, et al. cAMP-dependent phosphorylation sites and macroscopic activity of recombinant cardiac L-type calcium channels. Mol Cell Biochem. 1998;185(1/2):95–109. 9746216 10.1023/a:1006878106672

[31] De Jongh KS, Merrick DK, Catterall WA. Subunits of purified calcium channels: a 212-kDa form of alpha 1 and partial amino acid sequence of a phosphorylation site of an independent beta subunit. Proc Natl Acad Sci U S A. 1989;86(21):8585–8589. 2554320 10.1073/pnas.86.21.8585 PMC298327

[32] De Jongh KS, Warner C, Colvin AA, et al. Characterization of the two size forms of the alpha 1 subunit of skeletal muscle L-type calcium channels. Proc Natl Acad Sci U S A. 1991;88(23):10778–10782. 1720551 10.1073/pnas.88.23.10778 PMC53014

[33] Hell JW, Yokoyama CT, Wong ST, et al. Differential phosphorylation of two size forms of the neuronal class C L-type calcium channel alpha 1 subunit. J Biol Chem. 1993;268(26):19451–19457. 8396138

[34] Hell JW, Westenbroek RE, Breeze LJ, et al. N-methyl-D-aspartate receptor-induced proteolytic conversion of postsynaptic class C L-type calcium channels in hippocampal neurons. Proc Natl Acad Sci U S A. 1996;93(8):3362–3367. 8622942 10.1073/pnas.93.8.3362 PMC39613

[35] De Jongh KS, Murphy BJ, Colvin AA, et al. Specific phosphorylation of a site in the full-length form of the alpha 1 subunit of the cardiac L-type calcium channel by adenosine 3ʹ,5ʹ-cyclic monophosphate-dependent protein kinase. Biochemistry. 1996;35:10392–10402. 8756695 10.1021/bi953023c

[36] Wei X, Neely A, Lacerda AE, et al. Modification of Ca2+ channel activity by deletions at the carboxyl terminus of the cardiac alpha 1 subunit. J Biol Chem. 1994;269(3):1635–1640. 7507480

[37] Fuller MD, Emrick MA, Sadilek M, et al. Molecular mechanism of calcium channel regulation in the fight-or-flight response. Sci Signal. 2010;3(141):ra70. 20876873 10.1126/scisignal.2001152 PMC3063709

[38] Katchman A, Yang L, Zakharov SI, et al. Proteolytic cleavage and PKA phosphorylation of α1C subunit are not required for adrenergic regulation of Ca V 1.2 in the heart. Proc Natl Acad Sci U S A. 2017;114(34):9194–9199. 28784807 10.1073/pnas.1706054114 PMC5576811

[39] Mikami A, Imoto K, Tanabe T, et al. Primary structure and functional expression of the cardiac dihydropyridine-sensitive calcium channel. Nature. 1989;340(6230):230–233. 2474130 10.1038/340230a0

[40] Leach RN, Brickley K, Norman RI. Cyclic AMP-dependent protein kinase phosphorylates residues in the C-terminal domain of the cardiac L-type calcium channel alpha1 subunit. Biochim Biophys Acta. 1996;1281(2):205–212. 8664319 10.1016/0005-2736(96)00013-2

[41] Takahashi E, Fukuda K, Miyoshi S, et al. Leukemia inhibitory factor activates cardiac L-Type Ca2+ channels via phosphorylation of serine 1829 in the rabbit Cav1.2 subunit. Circ Res. 2004;94:1242–1248. 15044319 10.1161/01.RES.0000126405.38858.BC

[42] Erxleben C, Gomez-Alegria C, Darden T, et al. Modulation of cardiac Ca(V)1.2 channels by dihydropyridine and phosphatase inhibitor requires Ser-1142 in the domain III pore loop. Proc Natl Acad Sci U S A. 2003;100(5):2929–2934. 12601159 10.1073/pnas.2628046100 PMC151443

[43] Mitterdorfer J, Froschmayr M, Grabner M, et al. Identification of PK-A phosphorylation sites in the carboxyl terminus of L-type calcium channel α 1 subunits †. Biochemistry. 1996;35(29):9400–9406. 8755718 10.1021/bi960683o

[44] Haase H, Bartel S, Karczewski P, et al. In-vivo phosphorylation of the cardiac L-type calcium channel beta-subunit in response to catecholamines. Mol Cell Biochem. 1996;163–164(1):99–106. 10.1007/BF00408645 8974044

[45] Bunemann M, Gerhardstein BL, Gao T, et al. Functional regulation of L-type calcium channels via protein kinase a-mediated phosphorylation of the β 2 subunit. J Biol Chem. 1999;274(48):33851–33854. 10567342 10.1074/jbc.274.48.33851

[46] Gerhardstein BL, Puri TS, Chien AJ, et al. Identification of the sites phosphorylated by cyclic AMP-dependent protein kinase on the β 2 subunit of L-type voltage-dependent calcium channels †. Biochemistry. 1999;38(32):10361–10370. 10441130 10.1021/bi990896o

[47] Ganesan AN, Maack C, Johns DC, et al. β-Adrenergic stimulation of L-type Ca 2+ channels in cardiac myocytes requires the distal carboxyl terminus of α 1C but not serine 1928. Circ Res. 2006;98(2):e11–8. 16397147 10.1161/01.RES.0000202692.23001.e2 PMC2692538

[48] Lemke T, Welling A, Christel CJ, et al. Unchanged β-Adrenergic stimulation of cardiac L-type calcium channels in Ca v 1.2 phosphorylation site S1928A mutant mice. J Biol Chem. 2008;283(50):34738–34744. 18829456 10.1074/jbc.M804981200 PMC3259877

[49] Miriyala J, Nguyen T, Yue DT, et al. Role of Ca V β subunits, and lack of functional reserve, in protein kinase a modulation of cardiac Ca V 1.2 channels. Circ Res. 2008;102(7):e54–64. 18356540 10.1161/CIRCRESAHA.108.171736

[50] Brandmayr J, Poomvanicha M, Domes K, et al. Deletion of the C-terminal phosphorylation sites in the cardiac β-subunit does not affect the basic β-adrenergic response of the heart and the Cav 1.2 channel. J Biol Chem. 2012;287(27):22584–22592. 22589548 10.1074/jbc.M112.366484 PMC3391128

[51] Haase H, Alvarez J, Petzhold D, et al. Ahnak is critical for cardiac Ca(V)1.2 calcium channel function and its beta-adrenergic regulation. Faseb J. 2005;19:1969–1977. 16319140 10.1096/fj.05-3997com

[52] Pankonien I, Alvarez JL, Doller A, et al. Ahnak1 is a tuneable modulator of cardiac Ca(v)1.2 calcium channel activity. J Muscle Res Cell Motil. 2011;32(4–5):281–290. 22038483 10.1007/s10974-011-9269-2

[53] Emrick MA, Sadilek M, Konoki K, et al. Beta-adrenergic-regulated phosphorylation of the skeletal muscle Ca(V)1.1 channel in the fight-or-flight response. Proc Natl Acad Sci U S A. 2010;107:18712–18717. 20937870 10.1073/pnas.1012384107 PMC2972969

[54] Yang L, Katchman A, Samad T, et al. beta-adrenergic regulation of the L-type Ca2+ channel does not require phosphorylation of alpha1C Ser1700. Circ Res. 2013;113:871–880. 23825359 10.1161/CIRCRESAHA.113.301926 PMC3864014

[55] Muth JN, Yamaguchi H, Mikala G, et al. Cardiac-specific overexpression of the alpha(1) subunit of the L-type voltage-dependent Ca(2+) channel in transgenic mice. Loss of isoproterenol-induced contraction. J Biol Chem. 1999;274:21503–21506. 10419451 10.1074/jbc.274.31.21503

[56] Fu Y, Westenbroek RE, Scheuer T, et al. Phosphorylation sites required for regulation of cardiac calcium channels in the fight-or-flight response. Proc Natl Acad Sci U S A. 2013;110(48):19621–19626. 24218620 10.1073/pnas.1319421110 PMC3845157

[57] Yang L, Dai DF, Yuan C, et al. Loss of beta-adrenergic-stimulated phosphorylation of CaV1.2 channels on Ser1700 leads to heart failure. Proc Natl Acad Sci U S A. 2016;113:E7976–E85. 27864509 10.1073/pnas.1617116113 PMC5150375

[58] Poomvanicha M, Matthes J, Domes K, et al. Beta-adrenergic regulation of the heart expressing the Ser1700A/Thr1704A mutated Cav1.2 channel. J Mol Cell Cardiol. 2017;111:10–16. 28778765 10.1016/j.yjmcc.2017.07.119

[59] Liu G, Papa A, Katchman AN, et al. Mechanism of adrenergic CaV1.2 stimulation revealed by proximity proteomics. Nature. 2020;577(7792):695–700. 31969708 10.1038/s41586-020-1947-z PMC7018383

[60] Yang L, Katchman A, Kushner J, et al. Cardiac CaV1.2 channels require beta subunits for beta-adrenergic-mediated modulation but not trafficking. J Clin Invest. 2019;129:647–658. 30422117 10.1172/JCI123878 PMC6355231

[61] Rhee HW, Zou P, Udeshi ND, et al. Proteomic mapping of mitochondria in living cells via spatially restricted enzymatic tagging. Science. 2013;339(6125):1328–1331. 23371551 10.1126/science.1230593 PMC3916822

[62] Hung V, Zou P, Rhee HW, et al. Proteomic mapping of the human mitochondrial intermembrane space in live cells via ratiometric APEX tagging. Mol Cell. 2014;55(2):332–341. 25002142 10.1016/j.molcel.2014.06.003 PMC4743503

[63] Paek J, Kalocsay M, Staus DP, et al. Multidimensional tracking of GPCR signaling via peroxidase-catalyzed proximity labeling. Cell. 2017;169(2):338–49 e11. 28388415 10.1016/j.cell.2017.03.028 PMC5514552

[64] Beguin P, Nagashima K, Gonoi T, et al. Regulation of Ca2+ channel expression at the cell surface by the small G-protein kir/Gem. Nature. 2001;411(6838):701–706. 11395774 10.1038/35079621

[65] Finlin BS, Crump SM, Satin J, et al. Regulation of voltage-gated calcium channel activity by the Rem and Rad GTPases. Proc Natl Acad Sci U S A. 2003;100(24):14469–14474. 14623965 10.1073/pnas.2437756100 PMC283615

[66] Manning JR, Yin G, Kaminski CN, et al. Rad GTPase deletion increases L-type calcium channel current leading to increased cardiac contraction. J Am Heart Assoc. 2013;2(6):e000459. 24334906 10.1161/JAHA.113.000459 PMC3886777

[67] Levitan BM, Manning JR, Withers CN, et al. Rad-deletion phenocopies tonic sympathetic stimulation of the heart. J Cardiovasc Transl Res. 2016;9(5–6):432–444. 27798760 10.1007/s12265-016-9716-y PMC5143207

[68] Wang G, Zhu X, Xie W, et al. Rad as a novel regulator of excitation-contraction coupling and beta-adrenergic signaling in heart. Circ Res. 2010;106(2):317–327. 19926875 10.1161/CIRCRESAHA.109.208272

[69] Xu X, Marx SO, Colecraft HM. Molecular mechanisms, and selective pharmacological rescue, of Rem-inhibited CaV1.2 channels in heart. Circ Res. 2010;107(5):620–630. 20616312 10.1161/CIRCRESAHA.110.224717 PMC2952418

[70] Moyers JS, Zhu J, Kahn CR. Effects of phosphorylation on function of the Rad GTPase. Biochem J. 1998;333(Pt 3):609–614. 9677319 10.1042/bj3330609 PMC1219623

[71] Yang T, Puckerin A, Colecraft HM. Distinct RGK GTPases differentially use alpha1- and auxiliary beta-binding-dependent mechanisms to inhibit CaV1.2/CaV2.2 channels. PLoS One. 2012;7:e37079. 22590648 10.1371/journal.pone.0037079 PMC3349659

[72] Puckerin AA, Chang DD, Shuja Z, et al. Engineering selectivity into RGK GTPase inhibition of voltage-dependent calcium channels. Proc Natl Acad Sci U S A. 2018;115(47):12051–12056. 30397133 10.1073/pnas.1811024115 PMC6255209

[73] Beguin P, Ng YJ, Krause C, et al. RGK small GTP-binding proteins interact with the nucleotide kinase domain of Ca 2+-channel β-subunits via an uncommon effector binding domain. J Biol Chem. 2007;282(15):11509–11520. 17303572 10.1074/jbc.M606423200

[74] Katz AM. Role of phosphorylation of the sarcoplasmic reticulum in the cardiac response to catecholamines. Eur Heart J. 1980;1(suppl 1):A:29–33. 10.1093/eurheartj/1.suppl_1.29 7023943

